# Phyto-pharmacological evaluation and characterization of the methanolic extract of the *Baccaurea motleyana* Müll. Arg. seed: promising insights into its therapeutic uses

**DOI:** 10.3389/fphar.2024.1359815

**Published:** 2024-02-29

**Authors:** Suriya Akter Shompa, Hasin Hasnat, Saima Jahan Riti, Md. Mirazul Islam, Farjahan Nur, Safaet Alam, Chuxiao Shao, Shuanghu Wang, Peiwu Geng, Abdullah Al Mamun

**Affiliations:** ^1^ Department of Pharmacy, School of Pharmaceutical Sciences, State University of Bangladesh, Dhaka, Bangladesh; ^2^ Drugs and Toxins Research Division, BCSIR Laboratories Rajshahi, Bangladesh Council of Scientific and Industrial Research, Rajshahi, Bangladesh; ^3^ Central Laboratory of The Sixth Affiliated Hospital of Wenzhou Medical University, Lishui People’s Hospital, Lishui, Zhejiang, China

**Keywords:** *Baccaurea motleyana*, GC–MS, phytochemical, cytotoxic, antimicrobial, hypoglycemic, antidiarrheal, antidepressant

## Abstract

**Introduction:** Plants and their extracts have been integral to the development of medicinal treatments throughout history, offering a vast array of compounds for innovative therapies. *Baccaurea motleyana* Müll. Arg., commonly known as Rambai, is an evergreen tree with economic importance in the Old-World Tropics.

**Method:** The study investigates its phytochemical composition through Gas Chromatography-Mass Spectrometry (GC-MS) and evaluates its pharmacological properties, including antidiabetic, antidiarrheal, antimicrobial, and antidepressant effects.

**Result and Discussion:** The GC-MS analysis revealed 15 bioactive compounds in the methanol extract, with Phenol, 3,5-bis(1,1-dimethylethyl)-, Methyl stearate, and Hexadecanoic acid, methyl ester being the predominant ones. The cytotoxicity assay demonstrated significant activity in the ethyl acetate fraction. Antimicrobial assays indicated mild to moderate antibacterial activity. *In vivo* studies on mice revealed significant hypoglycemic, antidiarrheal, and antidepressant properties. Molecular docking studies against EGFR, DHFR, GLUT-3, KOR, and MOA identified promising compounds with potential therapeutic effects. The identified compounds exhibited favorable ADME/T properties, emphasizing their potential for drug development. The study underscores the promising therapeutic potential of *Baccaurea motleyana*, showcasing its diverse bioactive compounds with significant medicinal properties.

**Conclusion:** These findings lay the groundwork for future research, emphasizing the exploration of *B. motleyana* as a source of natural remedies for addressing prevalent health conditions.

## Introduction

Plants, having played a pivotal role in the historical development of remarkable medicines, stand poised to contribute significantly to a diverse array of innovative treatments (Alam et al., 2021a; Zaman et al., 2023). According to the World Health Organization (WHO), 80% of the global population relies on traditional healthcare for primary health services (Islam et al., 2022). The exploration of naturally occurring compounds for novel medication development has been a comprehensive endeavor (Alam et al., 2021a). For over 5,000 years, plants have been harnessed for their therapeutic properties, serving as sources for antibiotics, antineoplastic agents, analgesics, cardioprotective compounds, and various other medicinal applications (Taher et al., 2023). Interestingly, plant-derived medicines are predicted to comprise 25% of drugs in developed countries and 80% in rapidly advancing nations like India and China. Despite 400,000 global secondary plant metabolites, only 10,000 have been identified, emphasizing the untapped potential of phytochemicals as vital sources for novel medications (Hasnat et al., 2023).

*Baccaurea motleyana* Müll. Arg., commonly known as rambai, belongs to a lesser-known group of plants with economic importance in the Old World tropics, specifically in regions spanning from India to the Pacific. This evergreen tree, widely distributed in Southeast Asia, including Malaysia, Indonesia, and Thailand, is renowned for its brownish-yellow, globose fruits with an edible aril (Nurmayani et al., 2021; Debnath et al., 2022). The fruit is known as rambi in the Philippines, mafaifarang (general), ramai, or lam-khae (pattani), and raa-maa tee-ku (narathiwat) in Thailand. In Assam, India, it is referred to as leteku, while in Bangladesh, it goes by the names latkan or “bubi” (Debnath et al., 2022). Rambai is rich in vitamins, minerals, and bioactive compounds like phenolic acids and flavonoids, and they are utilized in various forms, such as raw consumption, cooking, stewing, and processing into jams and wines (Lim and Lim, 2012). Cultivated in various Southeast Asian nations, rambai has an oval-shaped fruit that undergoes a color transformation upon ripening, offering versatility in culinary applications (Lim and Lim, 2012; Nurmayani et al., 2021). Additionally, rambai’s bark has a historical application as an eye medicine and a postpartum medication for mothers (Prodhan and Mridu, 2021).

Since ancient times, the plant component of *B. motleyana* has been utilized in traditional medicine to address various health conditions. This plant serves as a medicinal plant, with applications in treating eye and skin inflammation. Additionally, rambai’s ecological significance extends to being a food source for various animals, including birds, rodents, deer, monkeys, and orangutans (Ramayani, 2020). Evidence showed that *B. motleyana* exhibits diverse medicinal uses across various cultures. The inner bark is traditionally employed for treating eye inflammation, exhibiting antibacterial activity against *Staphylococcus aureus, Bacillus cereus, Bacillus subtilis, Proteus vulgaris,* and *Escherichia coli* (Mohamed et al., 1994; Lim and Lim, 2012). The bark is also utilized in postpartum care, included in concoctions for mothers after childbirth, and applied in skincare products for its soothing effects (Mohamed et al., 1994; Lim and Lim, 2012). Furthermore, it plays a role in women’s healthcare, addressing issues such as leucorrhea and menstrual decay (Sisillia, 2009). Other traditional uses include its application as a remedy for diarrhea, strep throat, malaria, and sleep disorders and as an antibacterial agent (Prodhan and Mridu, 2021).

Plant secondary metabolites, including alkaloids, flavonoids, and steroids, play a vital role in plant defense and are utilized in the pharmaceutical industry for therapeutic purposes (Jain et al., 2019). The isolation of morphine in 1806 marked a transformative era, highlighting the medicinal importance of these compounds with a history spanning over 4,000 years (Elshafie and Camele, 2023). Constituting over 30% of medicinal products, alkaloids, and flavonoids among them, they exhibit diverse structural and therapeutic properties, making them valuable candidates for drug development (Twaij and Hasan, 2022). Gas chromatography–mass spectrometry (GC–MS) emerges as a pivotal technique for the effective separation and characterization of plant metabolites, contributing significantly to medicinal plant analysis and herbal drug validation (Iordache et al., 2009; Al-rubaye et al., 2017).

Chronic diseases such as diabetes mellitus (DM) continue to be a burden on millions of people worldwide in the face of global health challenges (Singab et al., 2014). Due to the drawbacks and adverse effects of current antidiabetic drugs, researchers are looking into alternative approaches. Medicinal plants have emerged as one such option for supplemental treatment that may have fewer side effects (Tafesse et al., 2017). However, in the case of gastrointestinal disorders, diarrhea poses a significant health concern, particularly in developing countries, leading to elevated rates of sickness and mortality (Bahekar and Kale, 2015). Traditional herbal medicine, endorsed by the World Health Organization (WHO), offers a viable approach to managing diarrheal disorders, presenting a bridge between traditional and modern healthcare (Atta and Mouneir, 2004). The unveiling of cancer’s molecular foundations over the last two decades has revolutionized treatment approaches, with recognition of the remarkable similarity in the fundamental processes across diverse tumor types (Weinberg, 1996). However, neurotoxicity associated with chemotherapy remains a critical limitation, necessitating a deeper understanding to guide appropriate therapeutic strategies (Schiff et al., 2009). Shifting to mental health, depression stands out as a predominant global disorder, affecting a substantial portion of the population (Santosh et al., 2011). The continuous difficulties in treating this serious medical issue are reflected in the search for safe and efficient antidepressant medications (Yu et al., 2002).

Despite the availability of synthetic drugs, the call for new therapeutics with fewer side effects resonates (Nisar et al., 2018). Clinical trials, grounded in the alignment between animal and clinical studies, play a pivotal role in advancing these discoveries (Alam et al., 2021b). In this all-encompassing approach, the investigation of plant-derived compounds stands as a continuum, advertising a potential worldview move in tending to changed wellbeing challenges over diabetes, gastrointestinal clutters, cancer, and mental wellbeing.

Exploring the therapeutic possibilities of *Baccaurea motleyana* Müll. Arg., an understudied plant with a rich history of traditional applications, this study aims to thoroughly examine the medicinal attributes of its seeds. The focus is on unraveling the historical uses and traditional applications of the plant to unlock its full medicinal potential. The study also intends to aid in the creation of new drugs by examining its medicinal qualities, locating bioactive components, and carrying out preclinical research. The investigation emphasizes the unrealized potential of compounds derived from plants and is in line with the world’s dependence on conventional healthcare. Preclinical studies with mice and *in silico* analyses will be conducted to validate and explore the pharmacological properties, laying the groundwork for the development of novel medications from this plant. Ultimately, this research has the noble goal of advancing natural sources for innovative treatments, addressing health challenges with potentially fewer side effects, and broadening pharmaceutical options.

## Materials and methods

### Collection of the plant

**FIGURE 1:**
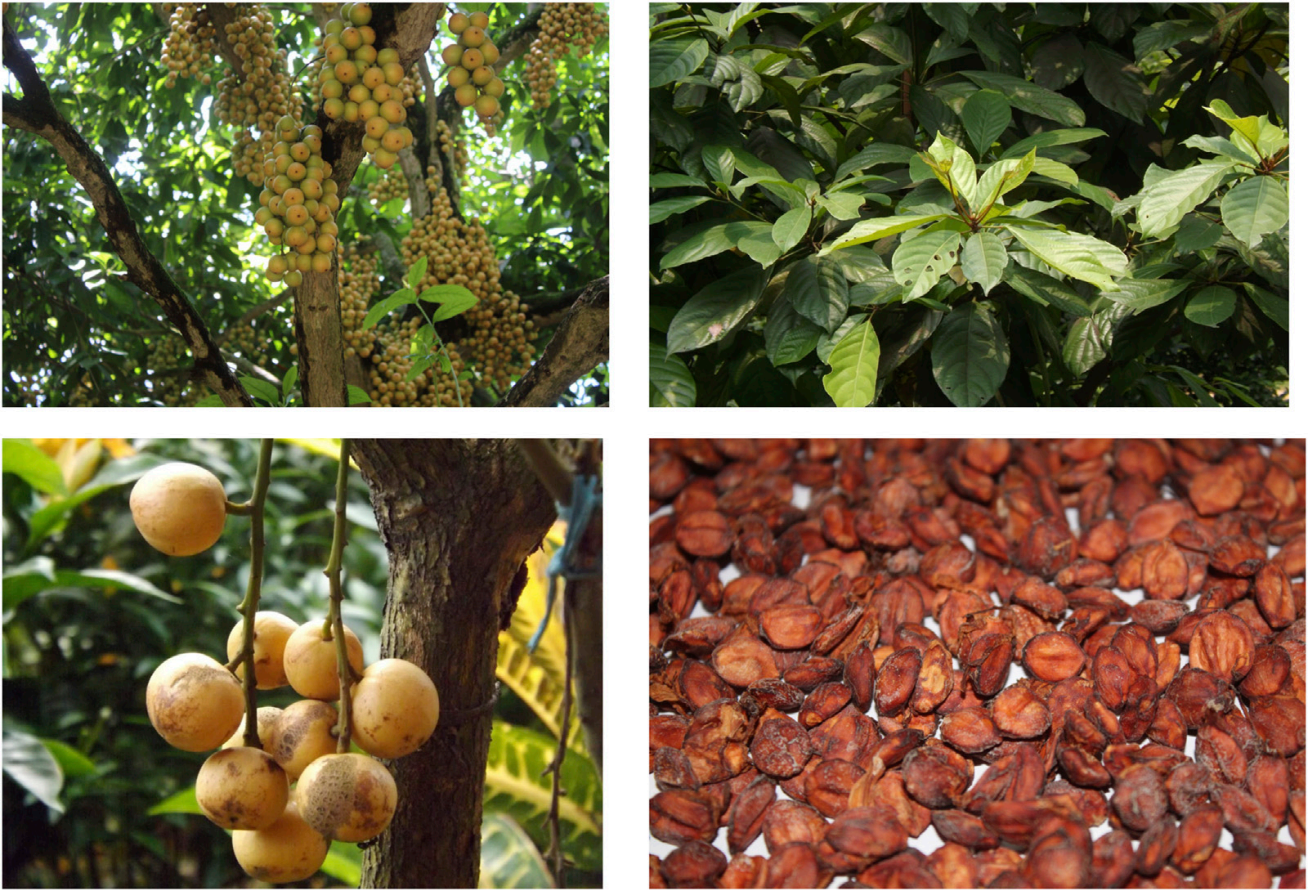
Plant and plant parts of *B. motleyana.*

In July 2022, seeds of *Baccaurea motleyana* were gathered from Narsingdi District, Dhaka Division, Bangladesh (Figure 1). The plant’s identification was validated by experts at the Bangladesh National Herbarium in Mirpur, Dhaka. A voucher specimen of the plant, assigned the accession number 84584, has been archived in the herbarium for future reference.

### Extraction of plant materials

*Baccaurea motleyana* seeds were obtained from the wild, subjected to shade drying, and subsequently pulverized using a mechanical grinder. The required quantity of leaf powder was accurately weighed, placed in a flask, completely immersed in methanol, incubated for the duration of 15 days, and then subjected to filtration. Subsequently, the filtered extract underwent concentration using a rotary evaporator (Islam et al., 2020).

### Partition into different fractionated extractives

The modified Kupchan partitioning method (VanWagenen et al., 1993) was employed for solvent–solvent partitioning. The crude methanolic extract (CME) of *B. motleyana* seeds (BMS) underwent fractionation with petroleum ether (PET), dichloromethane (DCM), ethyl acetate (EA), and distilled water, respectively, with an increasing relative polarity index. Subsequent to this process, rotary evaporation was utilized to obtain the petroleum ether-soluble fraction (PSF, 2.38 g), dichloromethane-soluble fraction (DSF, 2.75 g), ethyl acetate-soluble fraction (ESF, 2.18 g), and aqueous-soluble fraction (ASF, 1.6 g).

### Phytochemical analysis

#### GC–MS analysis

The seeds of *Baccaurea motleyana* were used to obtain a crude methanolic extract, and electron impact ionization (EI) was employed to extract beneficial compounds. The analysis was carried out utilizing a SHIMADZU GC–MS QP-2020 instrument equipped with an auto-sampler (AOC-20s) and auto-injector (AOC-20i). The analysis utilized a SH Rxi 5MS Sill column (30 m × 0.25 mm; 0.25 μm), and helium was employed as the carrier gas with a flow pressure of 1.72 mL/min. The temperature of the oven followed a programmed sequence, starting at 80°C (held for 2.00 min and raised at 5°C/min), reaching 150°C (held for 5.00 min), and concluding at a final temperature of 280°C (held for 5.00 min). The injector operated at 220°C, the ion source at 280°C, and a 5.0 μL injection volume was used with a 50:1 split ratio in the splitless injection mode. Ionization mass spectrometric analysis was performed at 70 eV, covering the mass range from 45 m/z to 350 m/z over a 50.0-min period. The solvent cut time was 5.0 min, and the total run time was 55.0 min. Identification of the bioactive compounds relied on the retention time, MS fragment ions, and the percentage of these compounds calculated from the total peak area. Phytochemicals were identified by comparing their mass spectra with entries in NIST08s, NIST08, and NIST14 libraries. This approach facilitated the determination of the chemical names, structures, and molecular masses of the bioactive components (Kim et al., 2016; Obaidullah et al., 2021).

### Biological activity study

#### Evaluation of *in vitro* cytotoxicity

The cytotoxic activity of the seeds of *B. motleyana* was evaluated by the brine shrimp lethality method (Meyer et al., 1982). In brief, 4 mg of the test sample was dissolved in dimethyl sulfoxide (DMSO), and serial dilution was performed to obtain variable concentrations (400.0–0.781 μg/mL) of the solutions. Ten living nauplii (*Artemia salina*) present in simulated seawater were taken in each test tube having tested solutions. After incubation at 24°C at room temperature in the presence of light, the number of surviving nauplii was calculated. For this experiment, vincristine sulfate was used as a positive control, and the solvent dimethyl sulfoxide (DMSO) served as a negative control. The percent (%) mortality was calculated for each dilution by using the following formula:

$$\%\text{ of mortality}=\frac{Number\;of\;the\;nauplii\;death}{Number\;of\;nauplii\;taken}\times 100\;\%.$$

The cytotoxic activity of the plant extract was assessed as the median lethal concentration value (LC_50_ value), which was calculated from a plot of the % of non-living nauplii against the log concentration of plant extracts using the standard curve of the reference drug lapatinib.

#### Antimicrobial screening

The crude plant samples were tested for antimicrobial susceptibility by following the disc diffusion method (Bauer et al., 1996). Based on Rashid et al. (2023), the most prevalent pathogenic microorganisms have been selected for this screening. They include five Gram-positive bacteria (*Bacillus cereus*, *Bacillus megaterium*, *Bacillus subtilis*, *Staphylococcus aureus*, and *Sarcina lutea*), eight Gram-negative bacteria (*Salmonella paratyphi*, *Salmonella typhi*, *Vibrio parahaemolyticus*, *Escherichia coli*, *Vibrio mimicus*, *Shigella dysenteriae*, *Pseudomonas aeruginosa*, and *Shigella boydii*), and three fungi (*Saccharomyces cerevisiae, Candida albicans,* and *Aspergillus niger*). Ciprofloxacin (5 μg/disc) and fluconazole (5 μg/disc) were used as reference drugs for antibacterial and antifungal activities, respectively. Shortly, nutrient agar plates were inoculated with a standardized inoculum of the test microorganisms. PDA media was used to influence the growth of fungi. Discs of approximately 6 mm diameter made of filter paper were loaded with test samples named BMS CME, BMS ASF, BMS PSF, BMS DSF, and BMS ESF and were evenly distributed on the surface of the nutrient media. The blank disc containing the solvent was used as the negative control. Approximately 24 h after the incubation at 37 °C (for bacteria) and 25 °C (for fungi) in the upright position, the zone of inhibition was recorded in millimeters, which shows the effectiveness of the plant samples against the microorganisms. The test was repeated three times, and the average diameter was taken.

#### Experimental animal

For the *in vivo* biological study, Swiss albino mice aged 4–5 weeks were collected from the Animal Resource Branch of the International Center for Diarrheal Diseases and Research, Bangladesh (ICDDR’B). They were fed ICDDR’B formulated rodent food and water (*ad libitum*) and kept in standard polypropylene cages under a regular laboratory environment with a 12-h light–dark cycle. Food was withdrawn before 12 h of the experiment. The experimental procedure conducted on the animals was approved by the Institutional Animal Ethics Committee (Zimmermann, 1983).

#### Experimental design for *in vivo* studies

Five mice groups were assigned for *in vivo* biological studies, with four mice in each group. Group I served as the negative control, receiving 1% Tween 80 in normal saline (10 mL/kg body weight). Group II served as the positive control and was administered with standard drugs for the respective experiments. Groups III, IV, and V received a crude methyl extract of *Baccaurea motleyana* at doses of 200 mg/kg, 400 mg/kg, and 600 mg/kg body weight of mice, respectively.

#### Hypoglycemic effect

The plant samples were subjected to tests to evaluate the hypoglycemic effect of the methanolic extract of the seeds of *Baccaurea motleyana* by the oral glucose tolerance test in mice (Islam et al., 2019). During the experiment, test samples at 200 mg/kg, 400 mg/kg, and 600 mg/kg doses were administered orally at 0 minute to all tested groups of mice. Glibenclamide (10 mg/kg of body weight) was used as the reference drug. After 30 min, all the mice were treated with a 10% glucose solution. The blood glucose level was recorded at 0 min and then at 30, 60, and 120 min using a glucometer. The percent reduction in the blood glucose level of *B. motleyana* was calculated by the equation below:

$$\%\text{ reduction in blood glucose}=\frac{{BG}_{control}-{BG}_{test}}{{BG}_{control}}\times 100\%,$$

where the average blood glucose level is expressed by BG_test_ for the test group and BG_control_ for the control group.

#### Antidiarrheal effect

The crude methanolic extract of *B. motleyana* seeds was subjected to tests to assess the antidiarrheal activity. The study was conducted by the method of castor oil-induced diarrhea in mice (Sisay et al., 2017). In brief, crude plant samples were administered at the respective doses (200, 400, and 600 mg per kg of body weight), and after 30 min, 0.5 mL of castor oil was injected to induce diarrhea in each mouse. Loperamide (50 mg/kg body weight) was used as the standard drug, and 1% Tween 80 in normal saline was used as the negative control. The antidiarrheal effect of the crude methanolic extract was observed for 4 h by a reduction in the frequency of defecation by the test samples. To evaluate the antidiarrheal activity, the following formula was used:

$$\%\text{ reduction in diarrhea}=\frac{D_{control}-D_{test}}{D_{control}}\times 100\%.$$

The average number of diarrheal defecation was expressed by D_test_ for the test group and D_control_ for the control group in the same duration.

#### CNS antidepressant activity

A diazepam-induced sleeping time test was carried out in Swiss albino mice to assess the CNS antidepressant activity of *B. motleyana* with a slight modification of the reference study (Akter et al., 2022). Fluoxetine (30 mg/kg body weight) was used as the reference drug. At first, plant samples at doses of 200, 400, and 600 mg per kg of body weight and standard were administered orally to each group of mice. After 30 min, each mouse was injected with diazepam (25 mg/kg body weight) through the intraperitoneal route to induce sleep. To evaluate the CNS antidepressant activity, the onset of sleeping time and the duration of sleeping time of each mouse were recorded.

### Molecular docking

#### Software

Utilizing a computer-based methodology, we evaluated the binding affinities of the compounds derived from the methanolic seed extract of *B. motleyana* against diverse target proteins. The analysis was conducted employing a range of software applications, including PyRx, PyMOL 2.3, Discovery Studio 4.5, and Swiss-PDB viewer, to comprehensively assess the molecular interactions.

#### Ligand preparation

The compounds listed in the table had their 3D SDF structures searched for and downloaded from PubChem (https://pubchem.ncbi.nlm.nih.gov/, accessed on 30 November 2023). Simultaneously, the 3D SDF structures of the five standard compounds lapatinib (PubChem CID_208,908), ciprofloxacin (PubChem CID_2764), glibenclamide (PubChem CID_3488), loperamide (PubChem CID_3955), and diazepam (PubChem CID_3016) were obtained from PubChem. A ligand library was then systematically generated by importing both the compounds and the standards into Discovery Studio 4.5. Subsequently, all the compounds were optimized using the PM6 semiempirical method, enhancing the precision of the docking process (Mahmud et al., 2021).

#### Target selection

We conducted computerized docking analysis on 15 compounds isolated from the methanolic extract of seeds of *B. motleyana* to investigate their potential cytotoxic, antimicrobial, hypoglycemic, antidiarrheal, and antidepressant properties. For cytotoxicity assessment, the 3D crystal structure of the epidermal growth factor receptor (EGFR) [PDB ID: 1XKK] (El Azab et al., 2021) was obtained from the Protein Data Bank (https://www.rcsb.org/ accessed on 30 November 2023). Similarly, the 3D structures of dihydrofolate reductase (DHFR) [PDB ID: 4M6J] (Khatun et al., 2021), glucose transporter 3 (GLUT3) [PDB ID: 4ZWB] (Mojica et al., 2017), kappa-opioid receptor (KOR) [PDB ID: 6VI4] (Alam et al., 2021b), and human monoamine oxidase A (MOA) [PDB: 2Z5X] (Rahman et al., 2020) were downloaded from the same source to evaluate their antimicrobial, hypoglycemic, antidiarrheal, and antidepressant activities, respectively.

#### Ligand–protein binding

The evaluation of affinities and potential binding patterns between the phytocompounds and target molecules was conducted through a computer-aided ligand–protein interaction diagram. The docking process utilized semi-flexible modeling, employing the advanced software application PyRx AutoDock Vina. Specific amino acids with their corresponding IDs were meticulously selected from the literature for individual receptors to ensure precise target docking. The protein underwent preparation by loading and formatting as the required macromolecule, ensuring exclusive binding of ligands to the intended targets.

To optimize docking against the selected macromolecules, SD files of ligands were imported and converted into pdbqt format using the Open Babel tool in PyRx AutoDock Vina software. Grid mapping defined active amino sites within grid boxes, maintaining a specified center and dimension axes, as detailed in Table 1. Default supportive functions were retained during this stage. Subsequently, a conclusive docking analysis was performed using AutoDock Vina (version 1.1.2) to ascertain the ligands’ affinity for the respective macromolecule. The final step involved result interpretation and the utilization of BIOVIA Discovery Studio version 4.5 to predict the most suitable 2D and 3D models.

**TABLE 1:** Selection of the target site and grid mapping of target receptors.

Receptor	Standard	Target binding sites	Reference	Grid box	
EGFR	Lapatinib	Leu 718, Val 726, Ala 743, Lys 745, Met 766, Lys 775, Arg 776, Leu 777, Leu 788, Thr 790, Gln 791, Leu 792, Met 793, Gly 796, Cys 797, Leu 799, Asp 800, Arg 803, Leu 844, Thr 854, Asp 855, and Phe 856	El Azab et al. (2021)	Center	x = 15.825187532
					y = 34.5721399187
					z = 35.800825226
				Dimension	x = 24.7838429695
					y = 19.8421893519
					z = 32.2648120992
DHFR	Ciprofloxacin	Ala 9, Ile 16, Lys 54, Lys 55, Thr 56, Leu 75, Ser 76, Arg 77, Glu 78, Arg 91, Ser 92, Leu 93, Gly 117, Ser 118, Ser 119, and Val 120	Khatun et al. (2021)	Center	x = 3.20608769545
					y = −3.56914211401
					z = −18.542188786
				Dimension	x = 20.3141281273
					y = 27.7715179138
					z = 27.0093471133
GLUT-3	Glibenclamide	Tyr 26, Thr 28, Gly 29, Val 30, Leu 167, Thr 191, Pro 194, Gln 198, Ile 309, Gly 312, Val 313, Thr 347, Trp 410, Leu 418, and Phe 42	Mojica et al. (2017)	Center	x = 111.281206841
					y = 14.4902327905
					z = 64.1163891831
				Dimension	x = 31.7208242373
					y = 35.5013767913
					z = 26.1685012591
KOR	Loperamide	Leu 103, Leu 107, Ser 136, Ile 137, Try 140, Ile 180, Trp 183, Leu 184, Ser 187, Ile 191, Leu 192 Ile 194, and Val 195	Alam M M et al. (2021)	Center	x = 54.1738452613
					y = −50.3649144833
					z = −16.3287029589
				Dimension	x = 15.1353410377
					y = 27.9195765234
					z = 17.9248494605
MOA	Diazepam	Ala 111, Phe 112, Pro 113, Tyr 121, Tyr 124, Asn 125, Trp 128, Arg 129, Thr 203, Thr 205, His 488, and Glu 492	Jendele et al. (2019)	Center	x = 50.7734380822
					y = 18.3788968615
					z = −22.1018219056
				Dimension	x = 17.607308401
					y = 19.3090003103
					z = 19.889121004

#### ADME/T study

In the field of computer-based drug design, there is a growing focus on conducting comprehensive pharmacokinetic studies that encompass absorption, distribution, metabolism, excretion, and toxicology. Evaluating drug-likeness through bioavailability studies has become integral to drug discovery efforts. ADMET analyses, crucial for deciphering pharmacological structures, can be accessed through resources like http://biosig.unimelb.edu.au/pkcsm/prediction. Widely utilized online platforms such as SwissADME (http://www.sib.swiss) predict drug-likeness based on the Lipinski rules and pharmacokinetic parameters. According to Lipinski, compounds meeting specific criteria, including a molecular weight below 500 amu, fewer than five hydrogen bond donor sites, fewer than 10 hydrogen bond acceptor sites, and a lipophilicity value (LogP) of ≤5, are considered orally accessible (Hasnat et al., 2023). The detailed results of ADMET and drug-likeness for these compounds are outlined in the provided table.

#### Statistical analysis

Statistical analysis was conducted utilizing GraphPad Prism 5.2 (GraphPad Software, Inc., La Jolla, CA, United States). The outcomes were presented as mean ± standard error (SEM). To assess statistical significance, one‐way analysis of variance (ANOVA) and Dunnett’s test were employed. Significance levels were denoted as *p < 0.5, **p < 0.01, and ***p < 0.001.

## Result

### GC–MS

**FIGURE 2:**
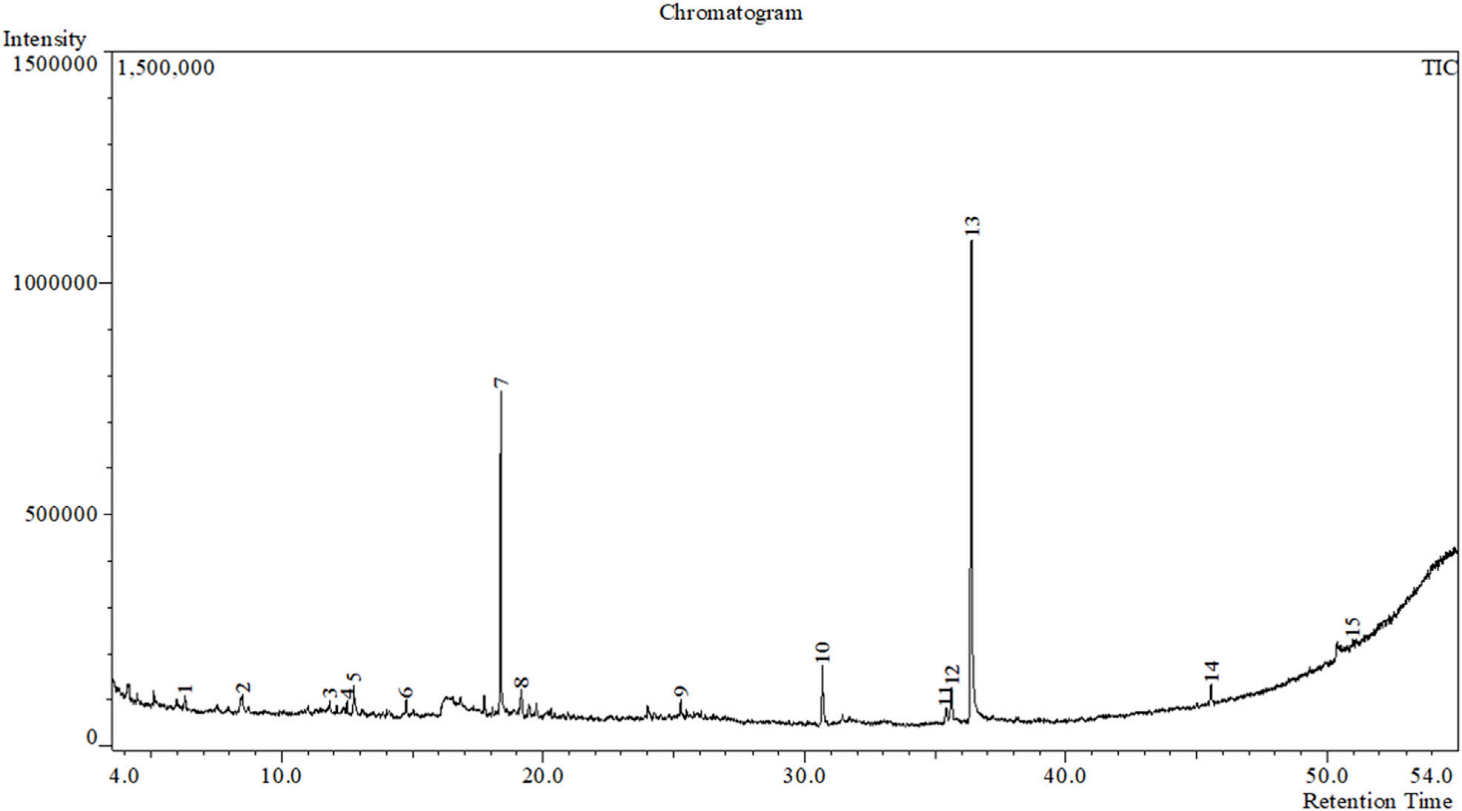
GC–MS chromatograph of the crude methanolic extract of seeds of *B. motleyana.*

In the GC–MS investigation, the examination of the crude methanolic extract from the seeds of *B. motleyana* uncovered 15 peaks, each signaling the presence of a bioactive molecule (Figure 2). The identification of these molecules involved a comparison of their molecular mass, chemical formula, and retention time with compounds acknowledged in the NIST library. This comprehensive analysis seeks to elucidate the diverse bioactive components found in the crude methanol seed extract of *B. motleyana*, offering valuable insights to augment the existing body of knowledge.

The distribution of compounds in the sample was quantified by representing their relative concentrations as peak area percentages. Predominant compounds, characterized by their high abundance, included phenol, 3,5-bis(1,1-dimethylethyl)- (45.257%), methyl stearate (21.251%), hexadecanoic acid, methyl ester (10.193%), dodecane, 2,6,11-trimethyl- (2.833%), cyclobarbital (2.711%), 9-octadecenoic acid (Z), methyl ester (2.697%), diisooctyl phthalate (2.491%), and benzaldehyde dimethyl acetal (2.486%). The remaining compounds were present in concentrations below 2%. Additional details, such as retention time, mass/charge ratio, and peak area, are provided in Table 2.

**TABLE 2:** GC–MS analysis of the methanolic extract of seeds of *B. motleyana*.

ID	Name	R.Time	m/z	Area	Concentration	Figure
1	Benzaldehyde dimethyl acetal	6.304	121.00	30424	2.486	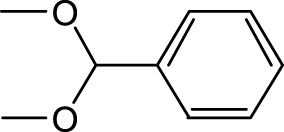
2	3-Tetradecene, (E)-	8.502	69.00	9807	0.801	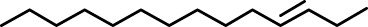
3	Oxalic acid, hexyl tridecyl ester	11.848	57.00	21828	1.783	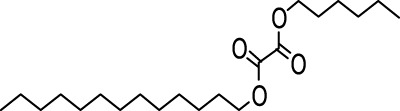
4	Dodecane, 2,6,10-trimethyl-	12.494	71.00	22750	1.859	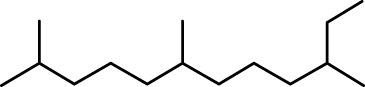
5	9-Methoxybicyclo[6.1.0]nona-2,4,6-triene	12.754	115.00	23846	1.948	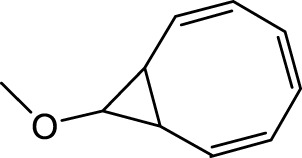
6	3-Hexadecene, (Z)-	14.763	69.00	16053	1.312	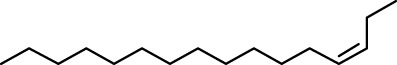
7	Phenol, 3,5-bis(1,1-dimethylethyl)-	18.370	191.00	553919	45.257	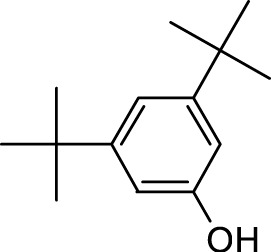
8	Dodecane, 2,6,11-trimethyl-	19.158	71.00	34671	2.833	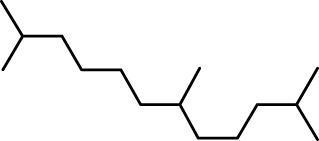
9	Hexadecane, 2,6,11,15-tetramethyl-	25.254	57.00	20521	1.677	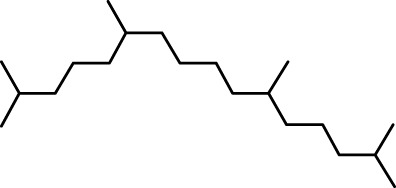
10	Hexadecanoic acid, methyl ester	30.675	74.00	124757	10.193	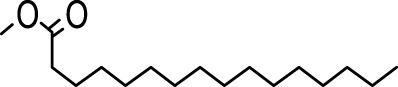
11	9,12-Octadecadienoic acid, methyl ester, (E,E)-	35.414	67.00	8595	0.702	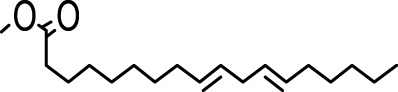
12	9-Octadecenoic acid (Z)-, methyl ester	35.624	55.00	33009	2.697	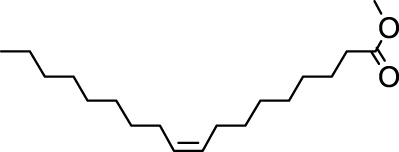
13	Methyl stearate	36.378	55.00	260098	21.251	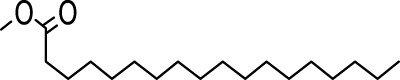
14	Diisooctyl phthalate	45.539	149.00	30488	2.491	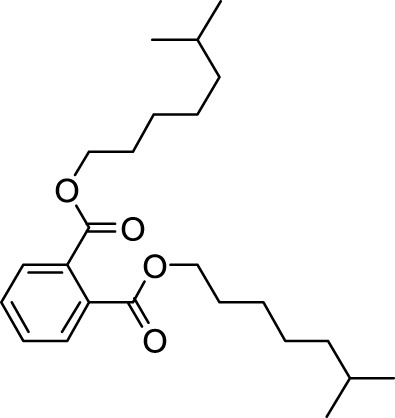
15	Cyclobarbital	50.949	207.00	33181	2.711	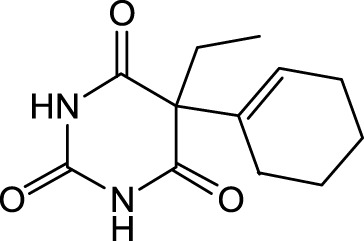

### Cytotoxic effect

**FIGURE 3:**
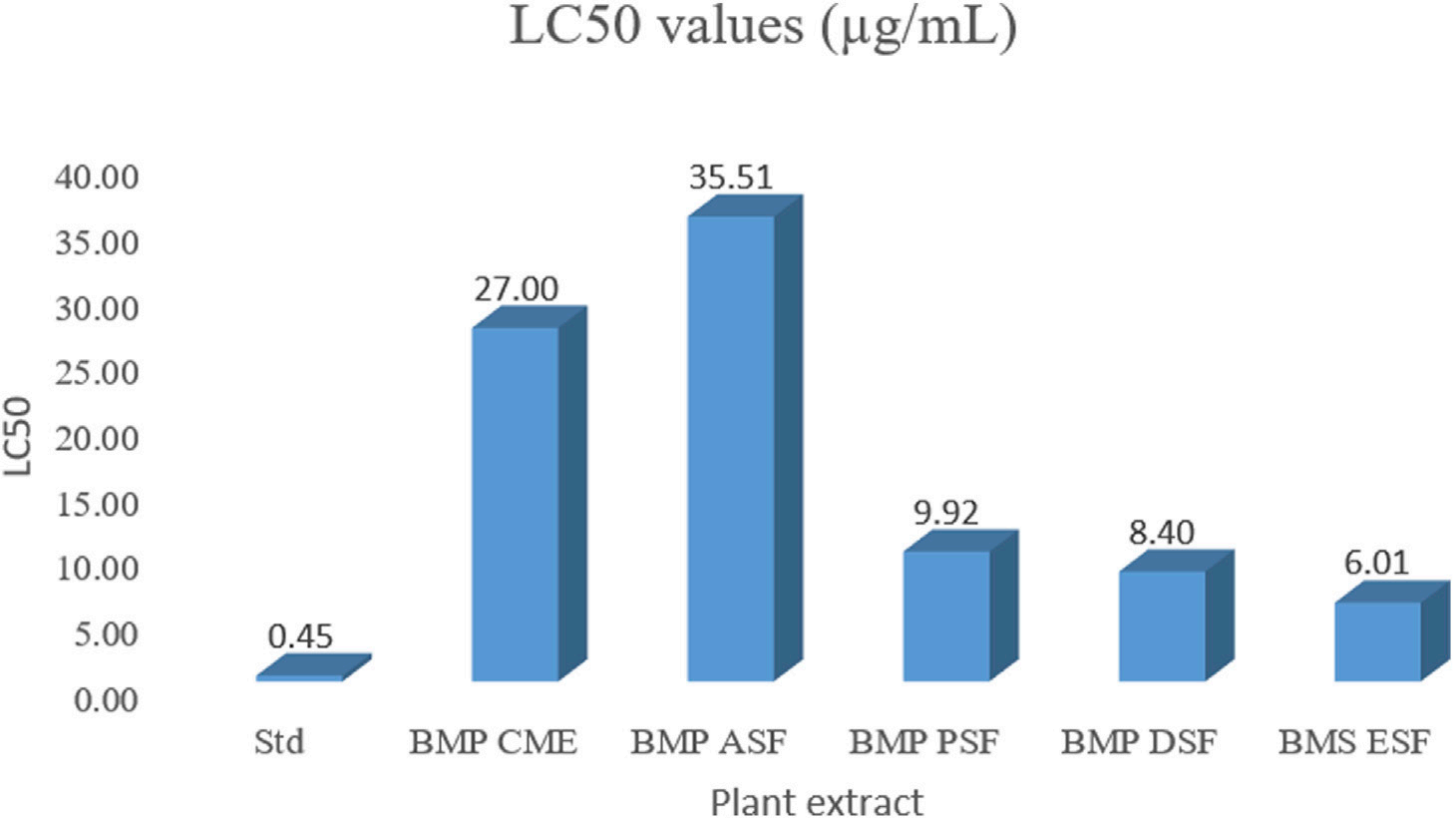
Cytotoxic effect of different fractions of the seed extract of *B. motleyana.*

The ethyl acetate (EA) fraction exhibited the most significant activity, displaying an LC_50_ value of 6.01 μg/mL, followed by the petroleum ether (PET) and dichloromethane (DCM) fractions with LC_50_ values of 9.92 and 8.40 μg/mL, respectively. In contrast, the crude methanolic extract (CME) and aqueous soluble fraction (ASF) demonstrated relatively weaker activity, with LC_50_ values of 27.00 and 35.51 μg/mL, respectively (Figure 3).

### Antimicrobial effect

**FIGURE 4:**
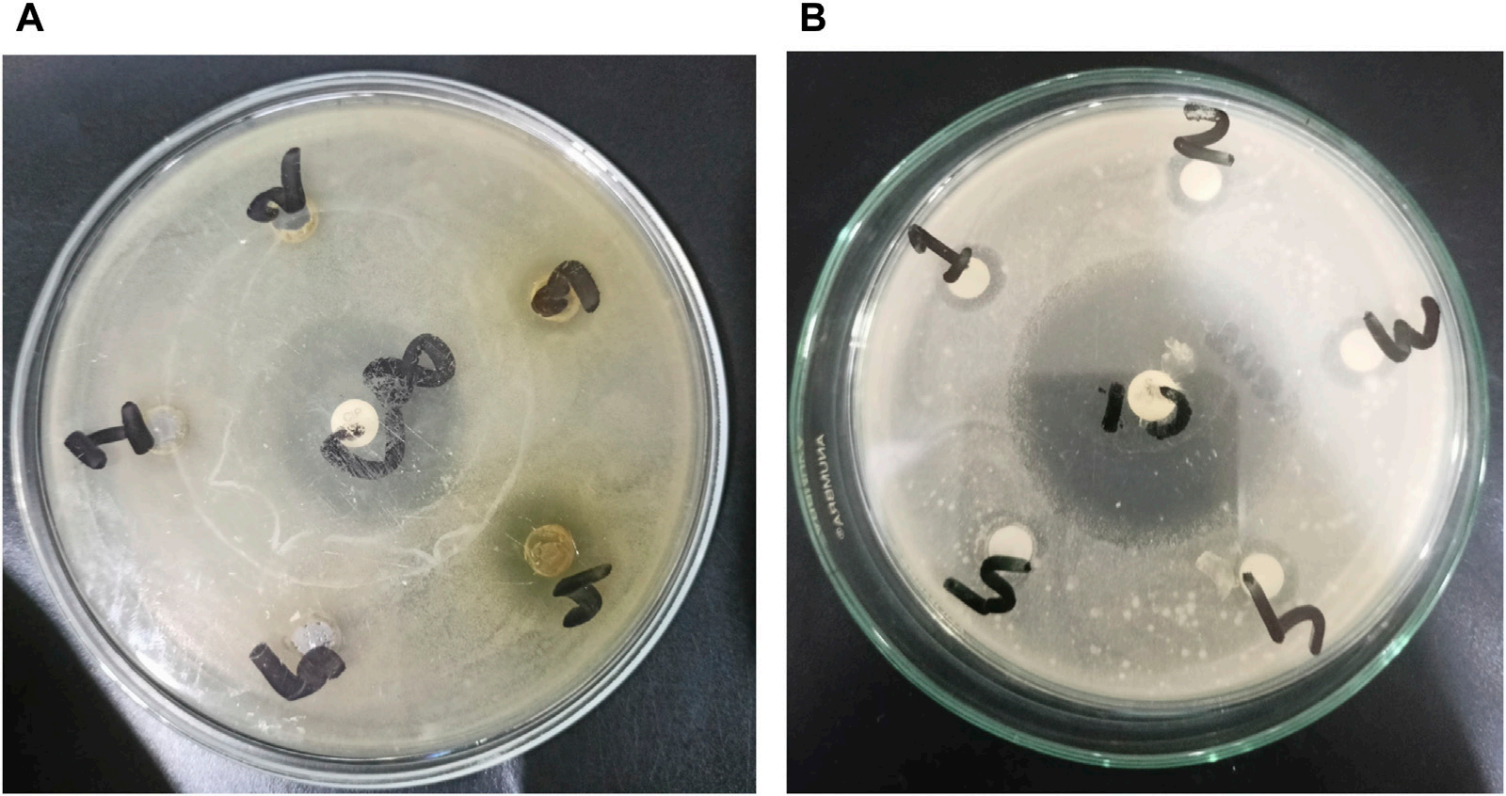
Development of zones of inhibition against microorganisms. In the case of Gram-negative *Escherichia coli*, activities are represented by **(A)**, with 1, 2, 3, 4, and 5 corresponding to BMS ASF, BMS PSF, BMS ESF, BMS CME, and BMS DSF, respectively. For Gram-positive *Staphylococcus aureus*, **(B)** represents the activity, where 1, 2, 3, 4, and 5 correspond to BMS CME, BMS DSF, BMS ESF, BMS ASF, and BMS PSF, respectively.

In this study, the antimicrobial assay was performed to evaluate the capacity of crude extracts to fight against the pathogenic microbes. The crude methanolic extract (BMS CSF), dichloromethane-soluble fraction (BMS DSF), and ethyl acetate-soluble fraction (BMS ESF) of seeds of *B. motleyana* showed mild-to-moderate antibacterial activity against both Gram-positive and Gram-negative bacteria compared with standard ciprofloxacin (Figure 4, Table 3).

**TABLE 3:** Antimicrobial activity displayed by different solvent soluble fractions of seeds of *B. motleyana*.

Test microorganism	Diameter of the zone of inhibition (mean ± SEM; mm)					
	BMS CME	BMS ASF	BMS PSF	BMS DSF	BMS ESF	Ciprofloxacin
Gram-positive bacteria						
*Bacillus cereus*	9 ± 0.05	0	0	0	7 ± 0.05	42 ± 0.08
*Bacillus megaterium*	6.99 ± 0.06	0	0	8 ± 0.05	8.99 ± 0.03	30 ± 0.08
*Bacillus subtilis*	10.98 ± 0.09	0	0	0	7 ± 0.04	41 ± 0.09
*Staphylococcus aureus*	12 ± 0.08	0	0	10 ± 0.12	8 ± 0.06	44 ± 0.05
*Sarcina lutea*	7.98 ± 0.1	0	0	0	9 ± 0.08	40 ± 0.09
Gram-negative bacteria						
*Salmonella paratyphi*	11 ± 0.05	0	0	8 ± 0.08	12 ± 0.05	43 ± 0.12
*Salmonella typhi*	9 ± 0.08	0	0	8 ± 0.04	11.98 ± 0.07	43 ± 0.06
*Vibrio parahaemolyticus*	9.97 ± 0.04	0	0	7 ± 0.08	11 ± 0.06	42 ± 0.04
*Escherichia coli*	13 ± 0.08	0	0	0	8 ± 0.06	41 ± 0.1
*Vibrio mimicus*	11 ± 0.08	0	0	0	9 ± 0.03	38 ± 0.06
*Shigella dysenteriae*	9 ± 0.06	0	0	8.99 ± 0.05	11 ± 0.06	40 ± 0.05
*Pseudomonas aeruginosa*	12 ± 0.08	0	0	0	10 ± 0.06	35 ± 0.08
*Shigella boydii*	11 ± 0.05	0	0	9.98 ± 0.09	8 ± 0.02	40 ± 0.06
Fungi						
*Saccharomyces cerevisiae*	8 ± 0.09	0	0	6.98 ± 0.01	8 ± 0.06	38 ± 0.05
*Candida albicans*	12 ± 0.05	0	0	0	11 ± 0.05	42 ± 0.06
*Aspergillus niger*	8.98 ± 0.07	0	0	8 ± 0.02	10 ± 0.08	52 ± 0.08

### Hypoglycemic effect

The hypoglycemic activity of seeds of *B. motleyana* in mice is summarized in Table 4. The most substantial reduction in glucose levels was noted with the administration of BMS CME at a dose of 600 mg/kg, showing a value of 23.58% ± 0.18%, in comparison to the standard, which exhibited a reduction of 49.53% ± 0.58%. Both demonstrated highly significant results with a *p*-value less than 0.01. Particularly noteworthy was the extremely significant glucose level reduction of 36.42% ± 0.24% observed 1 hour after the administration of BMS CME at a dose of 600 mg/kg.

**TABLE 4:** Hypoglycemic activity of seeds of *B. motleyana*.

Treatment	% Reduction of the blood glucose level			
	Hour after administration of the plant sample/drug			
	0 h	.5 h	1 h	2 h
Standard (2 mg/kg)	28.85 ± 0.32*	3.49 ± 2.99	7.95 ± 1.73	49.53 ± 0.58**
BMS 200 mg/kg	17.31 ± 0.35	3.99 ± 0.71	22.85 ± 0.34**	6.13 ± 0.44
BMS 400 mg/kg	11.54 ± 0.32	23.19 ± 0.67*	23.84 ± 0.58*	11.79 ± 0.38
BMS 600 mg/kg	10.58 ± 0.18	13.47 ± 2.27	36.42 ± 0.24***	23.58 ± 0.18**

Values are expressed as mean ± SEM (*n* = 5); CTL, negative control; STD, positive control; ***p < 0.001, **p < 0.01, and *p < 0.05 compared to negative control.

### Antidiarrheal effect

The methanolic extract of seeds of *B. motleyana* was subjected to a castor oil-induced antidiarrheal test, and the data are shown in Table 5. Castor oil administered orally influenced the episode of diarrhea, which continued for the following 4 h in the control mice. The methanolic extract of seeds of *B. motleyana* displayed remarkable antidiarrheal activity by reducing the number of diarrheal feces in test animals. Regarding the reduction of diarrheal episodes, the most pronounced activity was observed with the 200 mg/kg extract, achieving a suppression percentage of 55.56 ± 0.71. In comparison, the standard exhibited a slightly higher suppression of 61.11% ± 0.63%, while the 600 mg/kg and 400 mg/kg doses demonstrated suppressions of 50.00% ± 0.48% and 22.22% ± 1.19%, respectively. Furthermore, after 4 h, both the standard and the 600 mg/kg dose showed highly significant results, whereas the 200 mg/kg dose exhibited a significant outcome.

**TABLE 5:** Antidiarrheal activity of the methanolic extract of seeds of *B. motleyana*.

Treatment	% Reduction in the frequency of the diarrheal episode			
	Hour after administration of the plant sample/drug			
	1 h	2 h	3 h	4 h
BMS 200 mg/kg	100.00 ± 0.00*	77.78 ± 0.50*	66.67 ± 0.25	55.56 ± 0.71*
BMS 400 mg/kg	50.00 ± 0.50	55.56 ± 0.41	55.56 ± 0.41	22.22 ± 1.19
BMS 600 mg/kg	100.00 ± 0.00*	100.00 ± 0.00**	100.00 ± 0.00**	50.00 ± 0.48**
BMS (50 mg/kg)	100.00 ± 0.00*	66.67 ± 0.25*	44.44 ± 0.48	61.11 ± 0.63**

Values are expressed as mean ± SEM (n = 5); Standard = positive control; ***p < 0.001, **p < 0.01, and *p < 0.05 compared to negative control.

### Antidepressant effect

**FIGURE 5:**
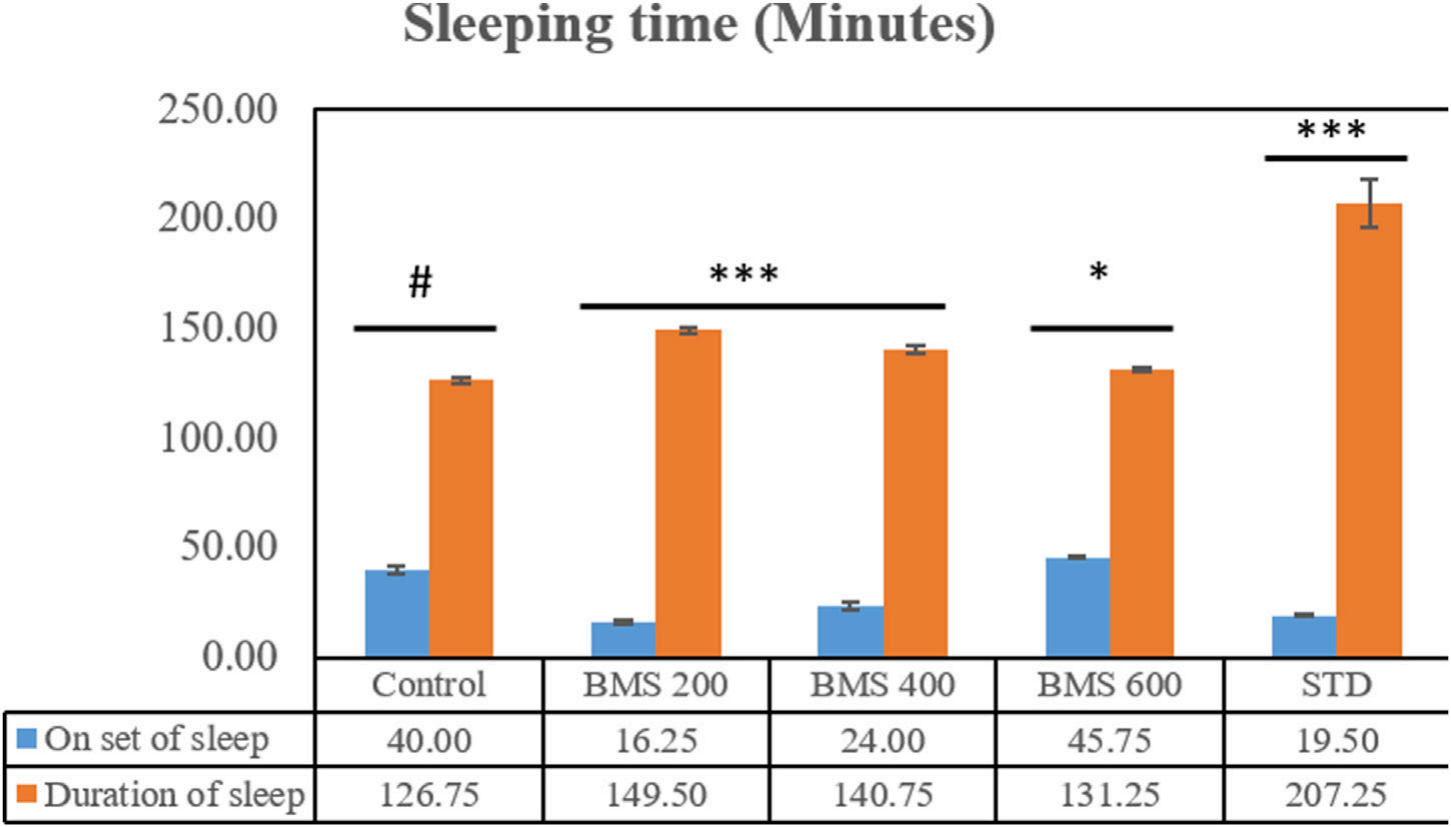
Onset and the duration of sleeping time displayed by the methanolic extract of seeds of *B. motleyana*.

The methanolic extract of seeds of *B. motleyana* at 200, 400, and 600 mg/kg doses were subjected to a diazepam-induced sleeping time test to observe the delay of the onset of sleeping time and reduction of sleep duration induced by diazepam in Swiss albino mice. The data found from the study are displayed in Figure 5. Surprisingly, the seed extract displayed a significant delay in sleep onset at 200 and 400 mg/kg doses, while the 600 mg/kg dose showed minimal impact (Table 6). However, doses of 200 and 400 mg/kg demonstrated an extended duration of sleeping time, suggesting dose-dependent effects on sleep Patterns (Table 7).

**TABLE 6:** Delay of the onset of sleeping time exhibited by the methanolic extract of seeds of *B. motleyana*.

	Onset of sleep (minutes)						
Group	M1	M2	M3	M4	Avg	SD	SEM
Control	28.000	32.000	30.000	70.000	40	20.067	10.033
BMS 200 mg/kg	20.000	6.000	18.000	21.000	16.25	6.946	3.473
BMS 400 mg/kg	24.000	29.000	28.000	15.000	24	6.377	3.189
BMS 600 mg/kg	17.000	118.000	24.000	24.000	45.75	48.280	186.000
STD	18.000	20.000	20.000	20.000	19.5	1.000	0.500

**TABLE 7:** Duration of sleeping time displayed by the methanolic extract of seeds of *B. motleyana*.

Group	Duration of sleep (minutes)						
	M1	M2	M3	M4	Avg	SD	SEM
Control	147	101	132	127	126.75	19.155	9.578
BMS 200 mg/kg	70	235	108	185	149.5	74.416	37.208
BMS 400 mg/kg	190	178	105	90	140.75	50.553	25.276
BMS 600 mg/kg	60	117	152	196	131.25	57.454	28.727
STD	222	218	176	213	207.25	21.156	10.578

### Molecular docking

The binding affinity of the identified compounds from the methanolic seed extract of B. motleyana against selected targets is shown in Table 8. Compound 14 displayed the lowest binding affinity toward EGFR, with a score of −7.3 kcal/mol, followed by compounds 7 and 15, which exhibited potential binding scores of −7 kcal/mol. However, when compared to the standard lapatinib with an affinity of −10.9 kcal/mol, seven other compounds scored lower than −6 kcal/mol. For DHFR, compound 15 demonstrated the highest activity, showing a notable binding affinity of −7.1 kcal/mol compared to the standard ciprofloxacin score of −8.2 kcal/mol. Additionally, compounds 7, 9, and 14 showed promising activity toward the receptor, with binding affinities of −6.3, −6, and −6.1 kcal/mol, respectively. In the case of GLUT-3, compounds 7 and 14 exhibited very prominent binding affinities of −7.6 and −7.9 kcal/mol, respectively, compared to the standard glibenclamide, with an affinity of −10.9 kcal/mol. Compounds 9 and 11 also showed satisfactory binding scores of −6.7 kcal/mol. Regarding **KOR**, compounds 7 and 14 approached the affinity score of the standard (−9.3 kcal/mol), demonstrating affinity scores of −7.1 and −7.6 kcal/mol, respectively. Additionally, compounds 9 and 15 conveyed prominent scores of −6.7 kcal/mol against the receptor. In comparison to the standard diazepam’s binding affinity toward **MOA**, which was −6.7 kcal/mol, compound 15 showed a very promising binding affinity of −6.6 kcal/mol. However, compounds 7, 9, and 14 demonstrated very promising affinities toward the receptor, with binding scores of −6.5, −6.3, and −6.4 kcal/mol, respectively.

**TABLE 8:** Binding affinities of identified compounds from the methanolic extract of *B. motleyana* seeds against EGFR, DHFR, GLUT-3, KOR, and MOA.

No.	Compound name	PubChem ID	Molecular weight (g/mol)	Binding affinities (kcal/mol)				
				EGFR (1xkk)	DHFR (4M6J)	GLUT3 (4ZWB)	KOR(6VI4)	MOA (2Z5X)
1	Benzaldehyde dimethyl acetal	62375	152.19	−5.6	−5	−5.5	−5.2	−5.2
2	3-Tetradecene, (E)-	5352802	196.37	−5.8	−4.8	−5.9	−5.7	−5.3
3	Oxalic acid, hexyl tridecyl ester	6420365	356.50	−5.9	−5.8	−6.5	−6.1	−5.9
4	Dodecane, 2,6,10-trimethyl-	19773	212.41	−6.1	−5.2	−6	−6.4	−5.5
5	9-Methoxybicyclo[6.1.0]nona-2,4,6-triene	5370526	148.20	−5.6	−5.5	−6.2	−6	−5.5
6	3-Hexadecene, (Z)-	5364494	224.42	−6	−4.8	−5.6	−6	−5.1
7	Phenol, 3,5-bis(1,1-dimethylethyl)-	70825	206.32	−7	−6.3	−7.6	−7.1	−6.5
8	Dodecane, 2,6,11-trimethyl-	35768	212.41	−6	−5.2	−6	−6.2	−6.1
9	Hexadecane, 2,6,11,15-tetramethyl-	136331	282.50	−6.7	−6	−6.7	−6.7	−6.3
10	Hexadecanoic acid, methyl ester	8181	270.50	−6.1	−5	−6.1	−5.8	−5.4
11	9,12-Octadecadienoic acid, methyl ester, (E,E)-	5362793	294.50	−6.5	−5.5	−6.7	−6.4	−5.8
12	9-Octadecenoic acid (Z)-, methyl ester	5364509	296.50	−6.4	−5.2	−6.2	−6.3	−5.8
13	Methyl stearate	8201	298.50	−6.1	−5	−5.9	−6	−5.6
14	Diisooctyl phthalate	33934	390.60	−7.3	−6.1	−7.9	−7.6	−6.4
15	Cyclobarbital	5,838	236.27	−7	−7.1	−6.2	−6.7	−6.6
Standards	Lapatinib	208908	581.10	−10.9	-	-	-	-
	Ciprofloxacin	2764	331.34	-	−8.2	-	-	-
	Glibenclamide	3488	494.00	-	-	−10.2	-	-
	Loperamide	3955	477.00	-	-	-	−9.3	-
	Diazepam	3016	284.74	-	-	-	-	−6.7

## Discussion

The search for innovative bioactive compounds with potential applications in emerging therapies places medicinal plants at the forefront. Consequently, there is a growing emphasis on the extensive utilization of plant-based natural remedies in developing nations, attracting considerable attention due to their multifaceted protective benefits and positive influences on human health. Traditional medicines, enjoying widespread popularity, are relied upon by approximately 80% of individuals, even in underdeveloped regions (Alam et al., 2020). Medicinal plant extracts constitute complex amalgamations of secondary metabolites derived from plants, animals, and microorganisms. Typically containing 10 to 60 ingredients in varying concentrations, these extracts often hinge on 2–4 key molecules for their biological features (Rahman et al., 2013). The exploration of the chemical composition and structure of samples reveals a myriad of biological potentials inherent in medicinal plant extracts.

Interestingly, there is a conspicuous lack of published research utilizing GC–MS/MS for the characterization of bioactive chemicals in the seeds of *Baccaurea motleyana*. In response to this gap, a meticulously planned investigation, involving GC–MS/MS evaluation, was initiated. In the crude methanolic seed extract of *B. motleyana*, the phenolic compound 3,5-bis(1,1-dimethylethyl) emerged as the most abundant. Recent studies have revealed that this compound, also known as 3,5-di-tert-butylphenol, possesses antifungal properties against *Candida* strains. It effectively hinders biofilm formation and impacts the viability of planktonic cells, inducing significant morphological changes in both planktonic and biofilm cells, particularly affecting the cell membrane integrity. Moreover, 3,5-di-tert-butylphenol demonstrated synergistic effects with sodium dodecyl sulfate, leading to further disruption of membrane integrity. The compound also initiates the production of endogenous radical oxygen species in *Candida*, contributing to its anti-biofilm activity (Vijayakumar and MuhilVannan, 2021). Additionally, this compound, along with its analogous 2,6-ditert-butylphenol or phenol, 2,6-bis(1,1-dimethylethyl), has been reported to exhibit a range of activities, including antioxidant, cytotoxic, insecticidal, and nematicidal, as well as antibacterial and antiviral properties (Zhao et al., 2020).

In our study, a high amount of methyl stearate, around 21.251%, was found in the BMC CME, which was previously known for its medicinal potency. For instance, methyl stearate, identified as a bioactive compound in the fermentation broth, plays a crucial role in inhibiting nematode infection. Through reducing egg hatching, repelling J2s from plant roots, and regulating essential parasitic nematode genes (Mi-flp-18 and 16D10), methyl stearate demonstrates significant efficacy in controlling *Meloidogyne incognita*. Moreover, its positive impact on promoting banana plant growth at lower concentrations highlights its potential as an eco-friendly strategy for nematode control, providing a promising alternative to conventional pesticides and contributing to sustainable agricultural practices (Lu et al., 2020).

Hexadecanoic acid methyl ester, the third most abounded bioactive compound (10.193%) from the extract, displayed significant antibacterial efficacy against *Staphylococcus aureus* W35, *Pseudomonas aeruginosa* D31, *Klebsiella pneumoniae* DF30, and *K. pneumoniae* B45. Also research identified volatile compounds in *Imperata cylindrica*, including hexadecanoic acid methyl ester with antibacterial effects against *P. aeruginosa*, *Bacillus subtilis*, and *K. pneumoniae* (Lalthanpuii and Lalchhandama, 2019). Studies on *Scenedesmus intermedius* also highlighted the inhibitory impact of the hexadecanoic acid methyl ester against Gram-positive and Gram-negative bacteria (Davoodbasha et al., 2018). As a fatty acid ester, the hexadecanoic acid methyl ester acts on bacterial cell membranes, disrupting energy production, inhibiting enzyme activity, and causing direct lysis, making it a promising antibacterial agent with both safety and efficacy (Shaaban et al., 2021). The wide range of bioactive substances found in our study, such as methyl stearate, hexadecanoic acid methyl ester, and 3,5-di-tert-butylphenol, highlights the enormous potential of medicinal plants. These compounds exhibit a wide range of activities, including antifungal, antibacterial, and nematode-inhibiting properties. As we combine conventional treatments with novel therapeutic approaches, these compounds offer exciting prospects for future research in the pharmaceutical domains.

The presence of cytotoxic compounds in plant material is often assessed through cytotoxicity assays, with brine shrimp serving as a common zoological specimen for such studies. The brine shrimp lethality test is validated for assessing cytotoxicity in human solid tumors and essential oils, identifying potent anticancer compounds (Niksic et al., 2021). In this assay, a lower LC_50_ value indicates higher toxicity. Extracts with LC_50_ values over 1,000 μg/mL are considered non-toxic, while those with LC_50_ between 500 and 1,000 μg/mL are weakly toxic (Apu et al., 2013). The ethyl acetate (EA) fraction from the *B. motleyana* seed extract demonstrated significant cytotoxic activity, presenting the lowest LC_50_ value of 6.01 μg/mL compared to the standard 0.45 μg/mL. Notably, PET and DCM fractions also exhibited promising activity with LC_50_ values of 9.92 and 8.40 μg/mL, respectively. However, it is essential to comment on the relatively poor activity observed in the remaining fractions when compared to the standard. These results establish the EA fraction from the *B. motleyana* seed extract as a viable candidate for additional investigation in the development of cytotoxic agents by highlighting its notable cytotoxic potential. The significant activity found in the dichloromethane DCM and PET fractions points to more research directions. For a thorough understanding of the extract’s cytotoxic qualities, it is crucial to isolate and characterize particular bioactive compounds, as highlighted by the relatively lower activity in other fractions.

The rise of infectious diseases poses a growing global threat, escalating the risk of antimicrobial resistance. This increasing trend not only endangers public health but also exacerbates the challenges associated with antimicrobial resistance on a global scale (Dhingra et al., 2020). WHO reported that Antimicrobial resistance (AMR) will cause approximately 10 million deaths 2050 (Murray et al., 2022). Therefore, it will be beneficial to develop new antibacterial drugs from nature that decrease the incidence of AMR by controlling the growth of bacteria. The result showed significant inhibitory effects against both Gram-positive and Gram-negative bacteria. The different fractions of plant samples displayed the most promising result with a range of zone of inhibition of 8–13 mm. The crude methanolic extract of seeds of *B. motleyana* exhibited the highest zone of inhibition against that of *E. coli* (13 mm), followed by *S. aureus* (12 mm). However, *C. albicans* was also found to be sensitive to the crude methanolic extract by displaying a zone of inhibition of 12 mm compared with standard fluconazole. The plant extracts exhibited antifungal properties that are comparable with the standard antifungal drug. However, this study might be helpful in studying the antimicrobial effect of the plant constituents more extensively and against other microorganisms so that they could be used to develop effective antimicrobial agents.

Diabetes is a chronic medical condition that is a global health challenge. As traditional plant sources are thought to have minimum side effects and are cost effective, various studies recommend them for the effective treatment of diabetes mellitus (Lee et al., 2021). In this experiment, the crude methanolic extract of plant samples at 600 mg/kg body wt. dose shows high hypoglycemic activity (36.42%) as compared to the standard drug glibenclamide (7.95%) 1 h later, as shown in Table 4. In comparison to *Baccurea motleyana*, other plants like *Berberis aristata* and *Bixa orellana* have demonstrated significant hypoglycemic effects, attributed to compounds like berberine and bixin, respectively. *Catharanthus roseus* displayed dose-dependent blood sugar-lowering activity, with isolated alkaloids showing potential for type 2 diabetes management (Alam et al., 2022). These findings highlight the diverse mechanisms and bioactive compounds present in different plant extracts, suggesting their potential in diabetes treatment.

Following castor oil ingestion, ricinoleic acid induces gut irritation and inflammation, disrupting gastrointestinal motility, secretions, and epithelial permeability. This disrupts the re-absorption of Na+, K+, and water, leading to the release of inflammatory mediators, such as prostaglandins, histamine, and nitric oxide (Alam et al., 2021b). In the present investigation, the methanolic extract of *B. motleyana* seeds demonstrated significant antidiarrheal effects at doses of 200 mg/kg and 600 mg/kg in a castor oil-induced diarrhea model, surpassing the efficacy of the reference drug loperamide. The study substantiates the notion that the extract effectively alleviates diarrhea by inhibiting hypersecretion and enteropooling, presenting potential therapeutic advantages (Billah et al., 2013). The antidiarrheal property of the tested plant samples could be due to phytochemicals like glycosides, alkaloids, tannins, terpenes, flavonoids, and other phenolic compounds present in *B. motleyana* (Gutiérrez et al., 2014). The extract demonstrated substantial reduction in intraluminal fluid accumulation, comparable to the widely prescribed antidiarrheal drug loperamide, suggesting its potential in managing diarrhea, possibly by inhibiting hypersecretion and enteropooling in the gut (Alam M M et al., 2021).

Although several treatment modalities have been developed for coronary artery disease, depression, and anxiety, full symptom relief without adverse effects is still unattainable. The imperfect pharmacokinetics and related side effects of current medications make it difficult to use them in clinical settings. As a result, there are now serious concerns about the safety, effectiveness, duration of action, and side effects of current medications. It is now essential to research new medications. Herbal medicine has emerged as a promising treatment option for these diseases because of the numerous neural targets that are involved (Fajemiroye et al., 2016). CNS stimulants have been used as a treatment option for depression (Avois et al., 2006). CNS stimulants generally alter sleep patterns by either shortening the duration of sleep, prolonging the onset of sleep, or affecting both aspects simultaneously (Rao and Tripathi, 2022). Among the three doses, the 600 mg/kg body weight dose exhibits the highest delay of onset of sleep (minutes), as shown in Figure 3, and the same dose displayed the lowest duration of sleeping time (Figure 5). Due to a significant correlation between reduced immobility and clinical potency in assessing antidepressants, the rat model is prioritized. Although the antidepressant test used in rats lacks symptomatology similar to human depression, it effectively distinguishes antidepressants from neuroleptics and anxiolytics. Determining the absolute effects of dopaminergic, anticholinergic, and GABAergic moieties as antidepressants is challenging (Emon et al., 2020). The methanolic extract of *B. motleyana* seeds demonstrates antidepressant-like effects, potentially restoring brain monoamines and reducing reserpine-induced depression in rats. Additional research has revealed the potential cytotoxic activity of the methanolic extract from *Baccaurea ramiflora* (Lour), suggesting a possible impact on the central nervous system through enhanced GABAergic inhibition. This effect parallels the actions of drugs with sedative–hypnotic, anxiolytic, and muscle relaxant properties, operating via the primary inhibitory neurotransmitter, gamma-aminobutyric acid (Nesa et al., 2018). However, the antidepressant action observed by plant extracts suggested the existence of a phytochemical constituent’s flavonoid (Hossain et al., 2009).

**FIGURE 6:**
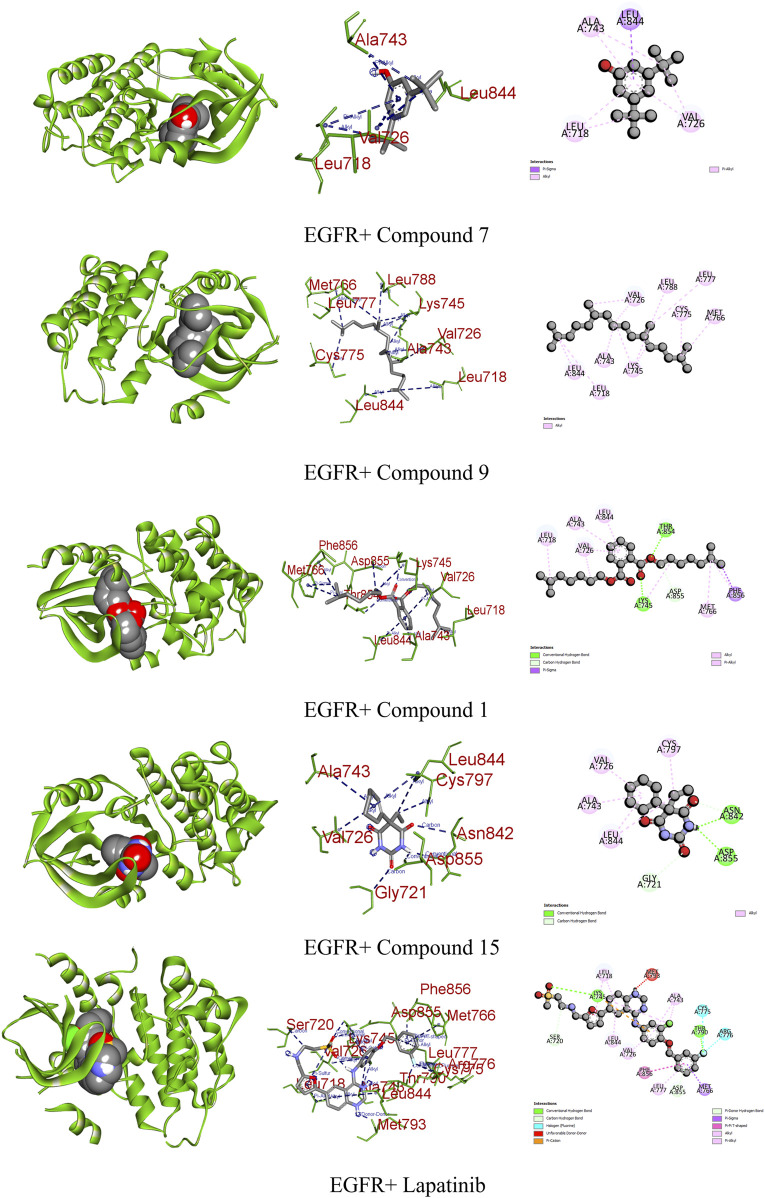
Graphical representation of the molecular interactions of the most prominent phytocompounds with the EGFR enzyme with 3D visualization.

EGFR plays a pivotal role in cancer management, particularly in the context of lung cancer. Overexpression of EGFR is implicated in various malignancies, including non-small-cell lung cancer. Upon ligand binding, EGFR undergoes conformational changes and phosphorylation, activating downstream pathways such as Raf1-extracellular signal-regulated kinase, PI3K/AKT, and STAT transcription factors. This activation contributes to uncontrolled cell proliferation and inhibits apoptosis, promoting cancer phenotypes (Ongko et al., 2022). Compound 14 demonstrated a significant binding affinity toward EGFR, forming one carbon–hydrogen, two conventional hydrogen, one pi–sigma, and five alkyl bonds with a potential binding score of −7.3 kcal/mol. Similarly, compounds 7 and 15 exhibited binding affinities of −7 kcal/mol. Compound 7 interacted through one pi–sigma and three pi-alkyl bonds, while compound 15 engaged through one carbon–hydrogen, two conventional hydrogens, and four alkyl bonds (Figure 6; Table 9).

**TABLE 9:** Bond and the binding site of highly active compounds against different targets, including EGFR, DHFR, GLUT-3, KOR, and MOA.

Receptor	Compound	Binding affinity (kcal/mol)	Bond type	Amino acids
EGFR	7	−7	Pi–sigma	Leu 844
			Pi–alkyl	Leu 718, Val 726, and Ala 743
	9	−6.7	Alkyl	Leu 718, Val 726, Ala 743, Lys 745, Met 766, Cys 775, Leu 777, Leu 788, and Leu 844
	14	−7.3	Carbon–hydrogen bond	Asp 855
			Conventional hydrogen bond	Lys 745 and Thr 854
			Pi–sigma	Phe 856
			Alkyl	Leu 718, Val 726, Ala 743, Met 766, and Leu 844
	15	−7	Carbon–hydrogen bond	Gly 721
			Conventional hydrogen bond	Asn 842 and Asp 855
			Alkyl	Val 726, Ala 743, Cys 797, and Leu 844
	Lapatinib	−10.9	Carbon–hydrogen bond	Ser 720 and Asp 855
			Conventional hydrogen bond	Lys 745 and Thr 790
			Pi–sigma	Met 766
			Alkyl	Leu 718, Val 726, Ala 743, Leu 777, and Leu 844
			Halogen(fluorine)	Cys 775 and Arg 776
			Unfavorable donor–donor	Met 793
			Pi–Pi T-shaped	Phe 856
DHFR	7	−6.3	Conventional hydrogen bond	Arg 91
			Pi–carbon	Arg 77
			Pi–alkyl	Leu 75
	9	−6	Alkyl	Val 8, Ala 9, Ile 16, Leu 22, Phe 34, and Tyr 121
	14	−6.1	Carbon–hydrogen bond	Ile 16, Gly 116, and Gly 117
			Conventional hydrogen bond	Thr 56 and Sern118
			Pi–pi T-shaped	Tyr 121
			Alkyl	Val 8, Leu 22, and Lys 55
	15	−7.1	Conventional hydrogen bond	Ile 7 and Glu 30
			Pi–sigma	Phe 34
			Alkyl	Ile 16 and Leu 22
	Ciprofloxacin	−8.2	Conventional hydrogen bond	Ala 9
			Carbon–hydrogen bond	Val 8, Thr 56, and Ser 118
			Pi–alkyl	Ile 16 and Leu 22
GLUT3	7	−7.6	Conventional hydrogen bond	Asn 315 and Glu 378
			Pi–pi T-shaped	Phe 289
			Alkyl	Ile 162, Ile 166, Ile 285, and Trp 386
	9	−6.7	Alkyl	Phe 24, Ile 166, Ile 285, Phe 289, Phe 377, and Trp 386
	11	−6.7	Conventional hydrogen bond	Asn 286
			Alkyl	Phe 24, Val 67, Ile 162, Ile 166, Ile 285, Phe 289, and Phe 377
	14	−7.9	Conventional hydrogen bond	Gln 281 and Asn 413
			Pi–pi T-shaped	Phe 289 and Phe 377
			Alkyl	Phe 24, Phe 70, Ile 166, Ile 285, and Trp 410
	Glibenclamide	−10.2	Conventional hydrogen bond	Asn 32, Val 67, and Asn 286
			Pi–sigma	Tyr 290
			Amide pi-stacked	Gly 417
			Alkyl	Ala 68, Ile 285, Phe 414, and Leu 418
KOR	C7	−7.1	Conventional hydrogen bond	Ile 180
			Pi–sigma	Trp 183 and Ile 191
			Alkyl	Val 195
	9	−6.7	Pi–sigma	Trp 183
			Alkyl	Phe 99, Ile 180, Leu 184, Ile 191, and Val 195
	14	−7.6	Conventional hydrogen bond	Ser 187
			Pi–pi stacked	Trp 183
			Alkyl	Phe 99, Tyr 140, Ile 180, Leu 184, Ile 191, and Val 195
	15	−6.7	Conventional hydrogen bond	Ser 187
			Carbon–hydrogen bond	SER 187
			Alkyl	Trp 183 and Ile 191
	Loperamide	−9.3	Pi-donor hydrogen bond	Ser 136
			Pi–sigma	Trp 183, Leu 184, and Ile 191
			Pi–pi T-shaped	Tyr 140
			Alkyl	Ile 180 and Val 195
MOA	7	−6.5	Pi–anion	Glu 492
			Pi–sigma	Phe 112
			Pi–alkyl	Trp 128 and His 488
	9	−6.3	Pi–sigma	His 488
			Alkyl	Phe 112, Tyr 121, Tyr 124, Trp 128, Arg 129, and Arg 493
	14	−6.4	Carbon–hydrogen bond	Asn 125 and His 488
			Conventional hydrogen bond	Thr 205
			Pi–anion	Glu 492
			Alkyl	Arg 129 and Arg 493
	15	−6.6	Conventional hydrogen bond	Phe 112 and Tyr 121
			Pi–alkyl	Tyr 124 and Trp 128
	Diazepam	−6.7	Pi–anion	Glu 492
			Pi–alkyl	Phe 112, Tyr 121, Trp 128, and His 488

**FIGURE 7:**
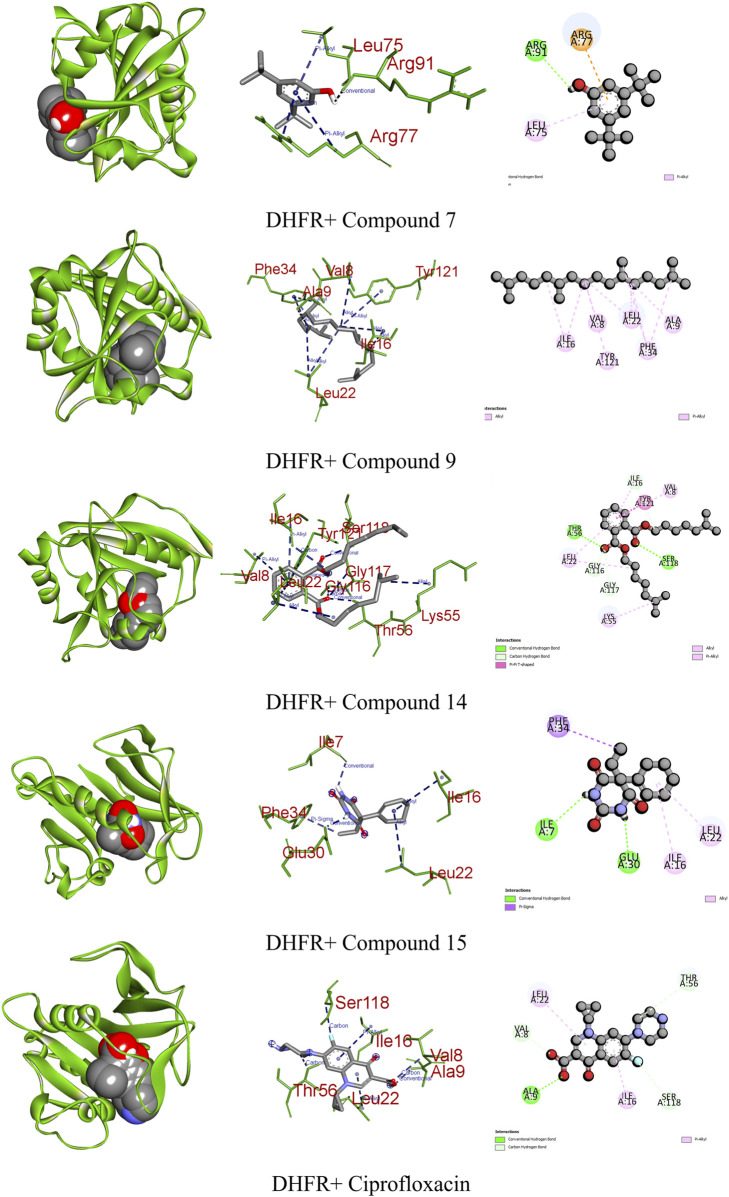
Graphical representation of the molecular interactions of the most prominent phytocompounds with the DHFR enzyme with 3D visualization.

DHFR, crucial in thymidylate biosynthesis, emerges as a promising target for infection treatment, with inhibitors potentially causing bacterial death (He et al., 2020). Additionally, DHFR-mediated disruptions in the folate pathway are implicated in uncontrolled cell growth, influencing cellular development and proliferation in malignancies (Kodidela et al., 2016). In computational docking studies, compound 15 displayed the highest binding affinity toward DHFR, disrupting two conventional hydrogens, one pi–sigma, and two alkyl bonds, resulting in a binding affinity score of −7.1 kcal/mol. This surpassed the standard ciprofloxacin (−8.2 kcal/mol), which formed one conventional hydrogen, three carbon–hydrogen, and two pi-alkyl bonds. Additionally, compounds 7, 9, and 14 showed satisfactory effects against the receptor, with all except compound 9 forming various types of interactions (Figure 7; Table 9).

**FIGURE 8:**
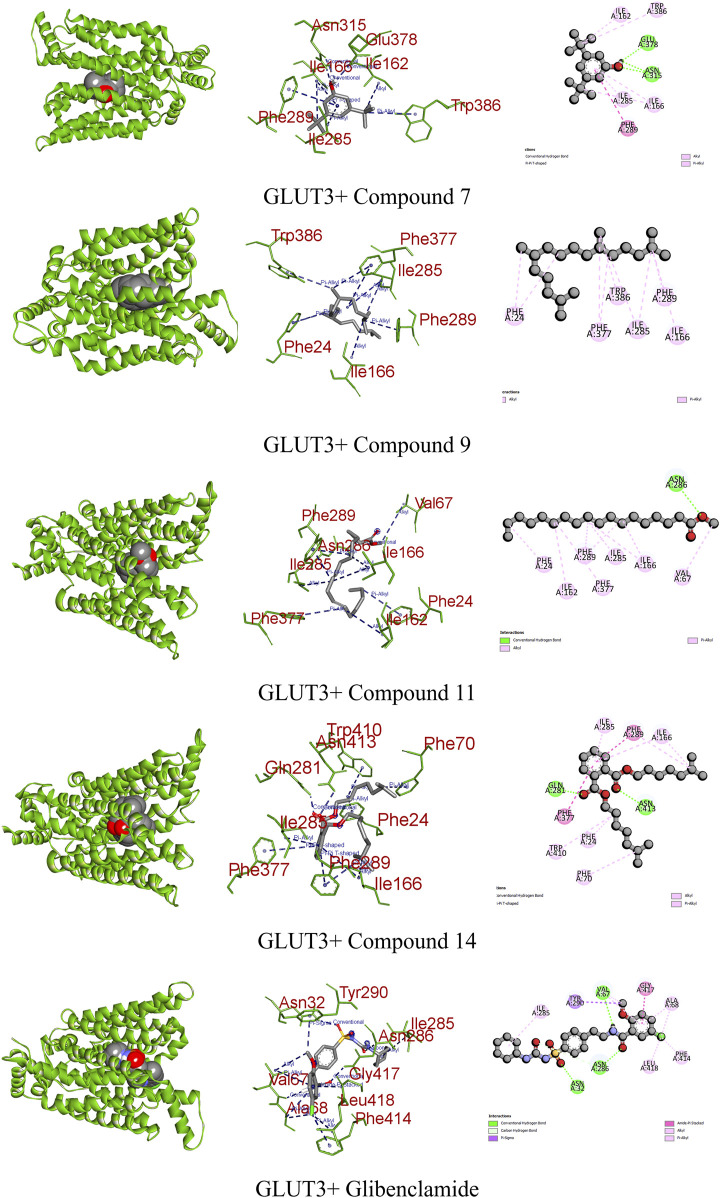
Graphical representation of the molecular interactions of the most prominent phytocompounds with the GLUT-3 enzyme with 3D visualization.

In diabetes, the regulation of glucose transporter isoforms, including GLUT-3, in retinal endothelial cells is pivotal. Elevated glucose levels lead to increased GLUT-3 mRNA expression, potentially enhancing the efficient utilization of glucose by retinal endothelial cells. Conversely, excessively high glucose concentrations result in the downregulation of glucose transporters, contributing to diabetic retinopathy by mediating damage in the retinal microvasculature. Understanding the regulatory mechanisms of GLUT-3 in response to varying glucose levels provides valuable insights for potential intervention therapies in diabetes-related complications (Knott et al., 1996). When interacting with GLUT-3, glibenclamide engaged the receptor through three conventional hydrogen bonds, a single pi–sigma, amide pi stacked, along with four alkyl bonds, resulting in a binding score of −10.2 kcal/mol. In contrast, compound 14 interacted with GLUT-3 through two conventional hydrogens, two pi–pi, and five alkyl bonds, showing a binding score of −7.9 kcal/mol. Similarly, compounds 7, 9, and 11 formed three, one, and two different types of interactions, respectively (Figure 8; Table 9).

**FIGURE 9:**
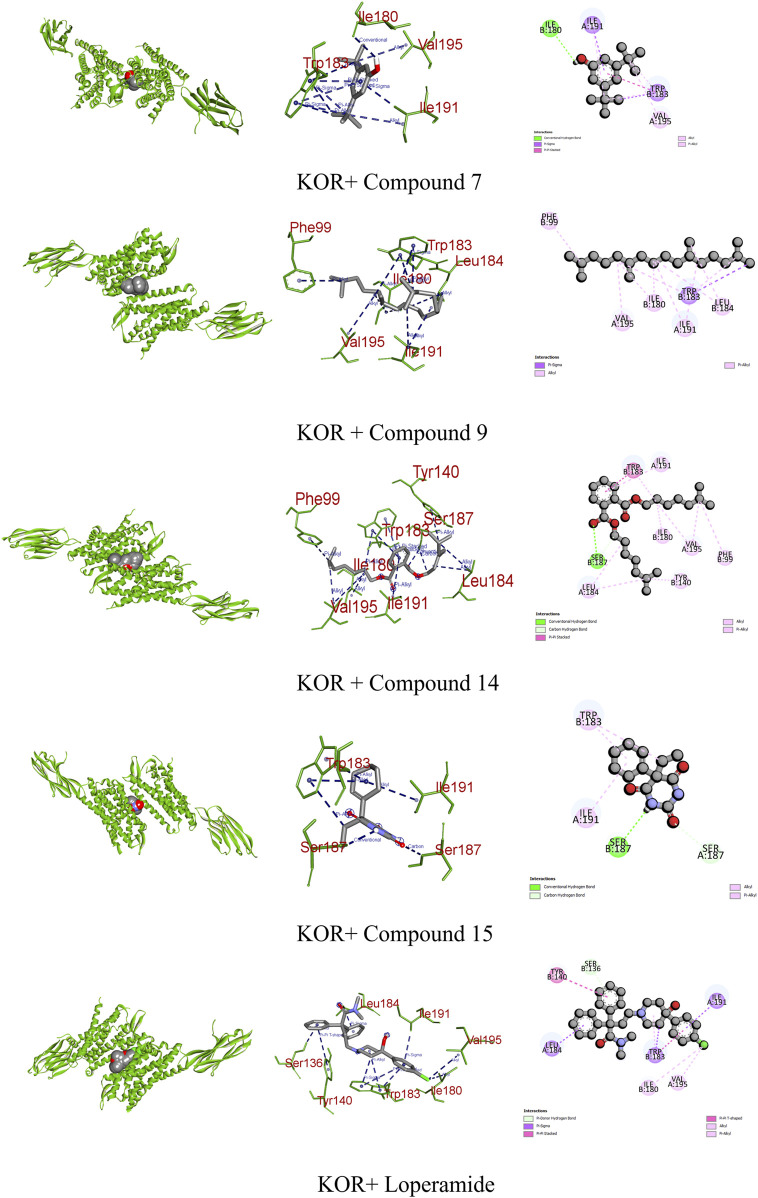
Graphical representation of the molecular interactions of the most prominent phytocompounds with the KOR enzyme with 3D visualization.

Opioid receptors, including µ, ƙ, and δ receptors, modulate gastrointestinal signaling by inhibiting enteric nerve activity and suppressing the neurotransmitter release, affecting excitatory and inhibitory motor pathways. This cascade results in delayed colonic transit, reduced enteric nerve excitability, and changes in secretion and fluid transport, ultimately impacting GI motility and stool consistency (Pannemans and Corsetti, 2018). Certain identified compounds, particularly compounds 7, 9, 14, and 15, demonstrated potential antioxidant activity by forming bonds with KOR. Compound 14 bound with a single conventional hydrogen, one pi–pi, and six alkyl bonds, resulting in a binding score of −7.6 kcal/mol compared to the standard score of −9.3 kcal/mol. Additionally, compounds 7, 9, and 15 formed three, two, and three different types of bonds, respectively, showing binding scores of −7.1, −6.7, and −6.7 kcal/mol (Figure 9; Table 9).

**FIGURE 10:**
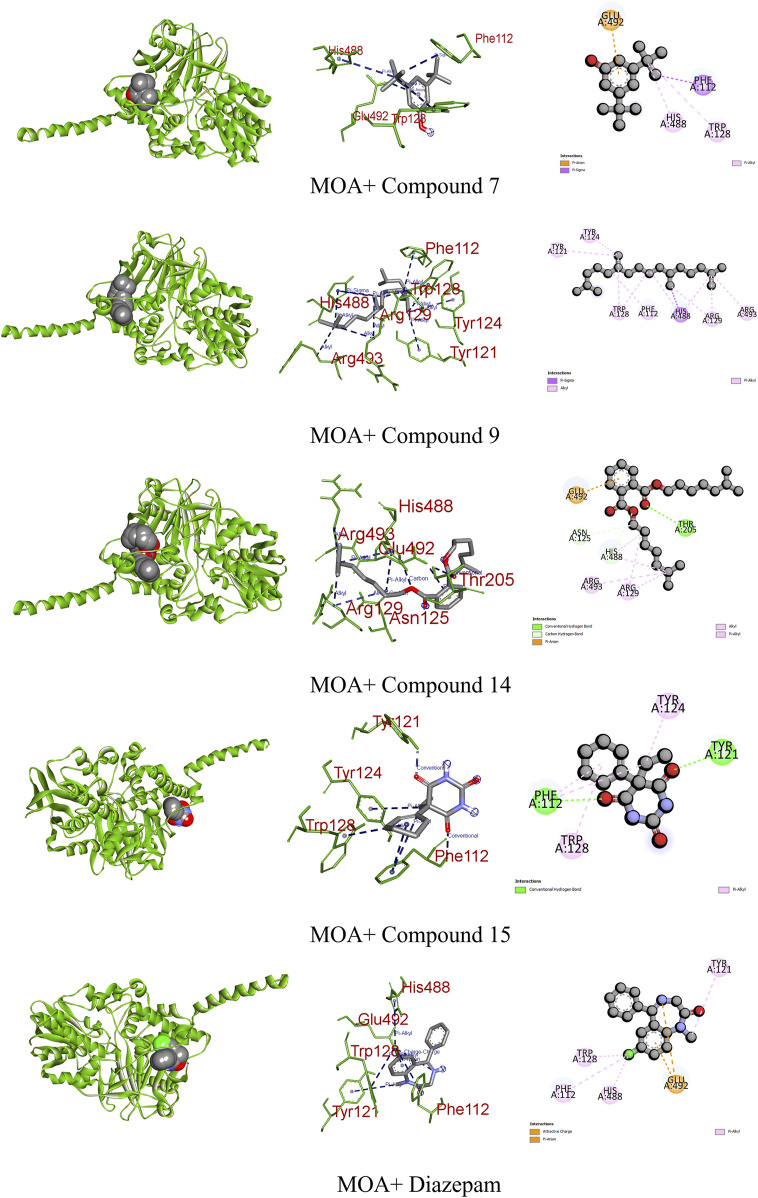
Graphical representation of the molecular interactions of the most prominent phytocompounds with the MOA enzyme with 3D visualization.

A study posits that monoamine oxidase A (MOA) density is likely elevated throughout the brain in individuals with major depressive disorder during untreated depressive episodes. This elevation is hypothesized as a potential mechanism contributing to the pathophysiology of depression as increased MOA activity could excessively lower brain monoamine levels, including serotonin, norepinephrine, and dopamine (Meyer et al., 2006). Regarding MOA, compounds 7, 9, 14, and 15 exhibited very strong binding affinities compared to the standard diazepam (−6.7 kcal/mol). Specifically, compound 15 formed two conventional hydrogen and two pi–alkyl bonds with the receptor, demonstrating the highest binding affinity of −6.6 kcal/mol. Meanwhile, diazepam formed one pi–anion and four pi–alkyl bonds (Figure 10; Table 9).

Surprisingly, our study revealed that various compounds identified from the methanolic extract of *B. motleyana* seeds displayed activity against multiple targets. This suggests a substantial involvement in mediating the diverse biological effects of the seed extract. This emphasizes the potential significance of these compounds in the overall pharmacological activity of the extract. Additionally, it is advisable to delve deeper into the exploration of these compounds in future research to gain a better understanding of their individual contributions and explore potential therapeutic applications.

The compounds with the highest binding affinities for the specified targets, as detailed in Table 10, underwent rigorous ADME/T screening. Notably, all these compounds exhibit robust intestinal absorptivity, coupled with negative water solubility, indicating their inherently lipophilic nature. In the term of the Rule of Five, compound 14 violates three rules, suggesting a limitation in terms of oral bioavailability. Conversely, compound 15 only violates a single rule, raising its candidacy as a potential oral drug. The remaining compounds each violate two rules. Despite these rule violations, all the compounds boast a remarkable bioavailability score of 0.55. Importantly, none of the compounds demonstrate AMES toxicity, underscoring their safety with respect to carcinogenicity. Similarly, all compounds test negative for hepatotoxicity. This comprehensive study strongly suggests that these compounds hold promising potential for future drug development.

**TABLE 10:** ADME/T study of best bounded compounds from the methanol seed extract of *B. motleyana* against EGFR, DHFR, GLUT-3, KOR, and MOA.

Property	Model name (Unit)	Compound 7	Compound 9	Compound 11	Compound 14	Compound 15
Absorption	Water solubility (log mol/L)	−3.876	−8.667	−7.343	−6.757	−2.636
	CaCo_2_ permeability (log Papp in 10–6 cm/s)	1.668	1.426	1.612	1.425	0.491
	Intestinal absorption (human) (% absorbed)	92.254	91.681	92.66	91.448	80.555
	Skin permeability (log Kp)	−2.364	−2.621	−2.719	−2.656	−3.78
	P-glycoprotein substrate	No	No	No	No	No
	P-glycoprotein I inhibitor	No	No	No	Yes	No
	P-glycoprotein II inhibitor	No	Yes	Yes	Yes	No
Distribution	VDss (human) (log L/kg)	0.545	0.595	0.272	0.194	0.009
	Fraction unbound (human) (Fu)	0.042	0	0.028	0	0.598
	BBB permeability	0.47	0.981	0.767	−0.184	0.052
	CNS permeability	−0.858	−0.982	−1.463	−2.169	−3.002
Metabolism	CYP2D6 substrate	No	No	No	No	No
	CYP3A4 substrate	Yes	Yes	Yes	Yes	No
	CYP1A2 inhibitor	Yes	Yes	Yes	No	No
	CYP2C19 inhibitor	No	No	No	No	No
	CYP2C9 inhibitor	No	No	No	No	No
	CYP2D6 inhibitor	No	No	No	No	No
	CYP3A4 inhibitor	No	No	No	No	No
Excretion	Total clearance (log mL/min/kg)	0.781	1.546	2.032	1.652	0.149
	Renal OCT2 substrate	No	No	No	No	No
Toxicity	AMES toxicity	No	No	No	No	No
	Max. tolerated dose (human) (log mg/kg/day)	0.409	0.062	−0.019	1.112	0.623
	hERG I inhibitor	No	No	No	No	No
	hERG II inhibitor	No	Yes	No	Yes	No
	Oral rat acute toxicity (LD_50_) (mol/kg)	2.346	1.522	1.617	1.249	2.569
	Oral rat chronic toxicity (LOAEL) (log mg/kg_bw/day)	1.736	1.273	3.004	2.695	1.022
	Hepatotoxicity	No	No	No	No	No
	Skin sensitization	Yes	Yes	Yes	No	No
	*T. pyriformis* toxicity (log ug/L)	1.667	1.322	1.603	0.664	0.257
	Minnow toxicity (log mM)	−0.108	−2.211	−1.6	−3.429	1.782
Drug-likeness	Bioavailability score (%)	0.55	0.55	0.55	0.55	0.55
	Lipinski’s Rule of Five	No; 2 violations: MW < 250, XLOGP3>3.5	No; 2 violations: Rotors>7, XLOGP3>3.5	No; 2 violations: Rotors>7, XLOGP3>3.5	No; 3 violations: MW > 350, Rotors>7, XLOGP3>3.5	No; 1 violation: MW < 250

The presence of the identified phytoconstituents in the seed extract of *B. motleyana* is implicated as a likely cause for a spectrum of biological effects, notably, antimicrobial, cytotoxic, antidiarrheal, anti-depressant, and antidiabetic activities. These findings underscore the multifaceted therapeutic potential of the plant’s seed extract. Furthermore, the ADME/T (absorption, distribution, metabolism, excretion, and toxicity) study strongly advocates for the plausible drug-like qualities of these phytochemicals. This points toward their potential candidacy as therapeutic agents with reduced side effects. These promising results suggest the necessity for more comprehensive research to unravel the full therapeutic potential of these phytoconstituents and explore their suitability for drug development.

### Limitation

Although this research has yielded promising findings, it is crucial to acknowledge the inherent limitations and approach them with a constructive perspective. First, the reliance on animal models, particularly rats, for assessing the antidepressant-like effects of the *B. motleyana* seed extract is a common and valuable starting point in preclinical studies. However, it is essential to recognize that animal models may not perfectly mirror human responses, emphasizing the need for cautious extrapolation to human applications. Exploring potential synergistic effects among the various compounds in the extract could enhance our understanding of the observed pharmacological activities. The observed antidiarrheal and hypoglycemic effects in animal models provide valuable insights, yet it is recognized that animal responses may not perfectly mirror human reactions. To address this, the call for rigorous human clinical trials is emphasized, ensuring a thorough evaluation of the extract’s efficacy, safety profiles, and optimal dosage regimens for potential therapeutic applications. The cytotoxicity assay using brine shrimp larvae, while being a widely used method, is acknowledged for its limitations in accurately predicting human cytotoxic responses. Recognizing this constraint highlights the importance of incorporating diverse cytotoxicity assays to obtain a more comprehensive understanding of the plant extract’s safety profile. In conclusion, while these limitations are acknowledged, they present opportunities for further research and refinement. Addressing these considerations will contribute to a more nuanced understanding of the potential therapeutic applications of the *B. motleyana* seed extract, laying the groundwork for future advancements in natural medicine. Additionally, the *in silico* data will help in boosting up the finding of potential leads for further deeper experiments to prove their drug candidacy. Overall, while the study provides valuable insights into the pharmacological potential of the *B. motleyana* seed extract, further research involving diverse experimental models, including human clinical trials, is necessary to validate and extend these findings.

## Conclusion

In summary, this comprehensive investigation elucidates the remarkable pharmacological versatility of *B. motleyana* seeds. The study underscores their substantial therapeutic potential, revealing significant bioactivities encompassing antidiarrheal, antimicrobial, antioxidant, hypoglycemic, and antidepressant properties. The identification of bioactive compounds through meticulous GC–MS analysis, coupled with insightful *in silico* studies, provides valuable insights into their potential molecular interactions with diverse therapeutic targets. Despite some rule violations in terms of oral bioavailability, the compounds exhibit promising ADME/T profiles, highlighting their candidacy for drug development. The multi-target activities of these compounds not only emphasize their integral role in the overall pharmacological effects of the seed extract but also open promising avenues for future research and pharmaceutical applications. This study contributes to the growing body of knowledge on plant-based remedies, emphasizing their potential as sources for novel and effective therapeutic agents.

## Data Availability

The raw data supporting the conclusions of this article will be made available by the authors, without undue reservation.
